# Intermediate hyperglycaemia, diabetes and blood pressure in rural Bangladesh: five-year post-randomisation follow-up of the DMagic cluster-randomised controlled trial

**DOI:** 10.1016/j.lansea.2022.100122

**Published:** 2022-12-10

**Authors:** Edward Fottrell, Carina King, Naveed Ahmed, Sanjit Kumer Shaha, Joanna Morrison, Malini Pires, Abdul Kuddus, Tasmin Nahar, Hassan Haghparast-Bidgoli, A.K. Azad Khan, Kishwar Azad

**Affiliations:** a UCL Institute for Global Health, University College London, London, UK; b Department of Global Public Health, Karolinska Institutet, Stockholm, Sweden; c Centre for Health Research & Implementation, Diabetic Association of Bangladesh, Dhaka, Bangladesh

**Keywords:** Diabetes, Bangladesh, Cardiovascular risk, Community intervention, Prevention, Control, Cluster randomised controlled trial, Rural

## Abstract

**Background:**

The DMagic trial showed that participatory learning and action (PLA) community mobilisation delivered through facilitated community groups, and mHealth voice messaging interventions improved diabetes knowledge in Bangladesh and the PLA intervention reduced diabetes occurrence. We assess intervention effects three years after intervention activities stopped.

**Methods:**

Five years post-randomisation, we conducted a cross-sectional survey among a random sample of adults aged ≥30-years living in the 96 DMagic villages, and a cohort of individuals identified with intermediate hyperglycaemia at the start of the DMagic trial in 2016. Primary outcomes were: 1) the combined prevalence of intermediate hyperglycaemia and diabetes; 2) five-year cumulative incidence of diabetes among the 2016 cohort of individuals with intermediate hyperglycaemia. Secondary outcomes were: weight, BMI, waist and hip circumferences, blood pressure, knowledge and behaviours. Primary analysis compared outcomes at the cluster level between intervention arms relative to control.

**Findings:**

Data were gathered from 1623 (82%) of the randomly selected adults and 1817 (87%) of the intermediate hyperglycaemia cohort. 2018 improvements in diabetes knowledge in mHealth clusters were no longer observable in 2021. Knowledge remains significantly higher in PLA clusters relative to control but no difference in primary outcomes of intermediate hyperglycaemia and diabetes prevalence (OR (95%CI) 1.23 (0.89, 1.70)) or five-year incidence of diabetes were observed (1.04 (0.78, 1.40)). Hypertension (0.73 (0.54, 0.97)) and hypertension control (2.77 (1.34, 5.75)) were improved in PLA clusters relative to control.

**Interpretation:**

PLA intervention effect on intermediate hyperglycaemia and diabetes was not sustained at 3 years after intervention end, but benefits in terms of blood pressure reduction were observed.

**Funding:**

10.13039/501100000265Medical Research Council UK: MR/M016501/1 (DMagic trial); MR/T023562/1 (DClare study), under the Global Alliance for Chronic Diseases (GACD) Diabetes and Scale-up Programmes, respectively.

**Research in context**

**Evidence before this study**

The Bangladesh DMagic trial reported in 2019 that mHealth and Participatory Learning and Action (PLA) community mobilisation interventions were effective at increasing knowledge and awareness of type-2 diabetes and its risk factors in rural Bangladesh. The PLA intervention also resulted in large reductions in population prevalence of diabetes and intermediate hyperglycaemia, and reductions in the incidence of type 2 diabetes among an intermediate hyperglycaemic cohort. Economic evaluation showed the PLA intervention to be highly cost effective and subsequent equity analysis showed that PLA impacts were observed across age, sex and wealth groups.

The medium- to long-term impacts of such population-level diabetes prevention and control interventions is unknown though evidence from other settings suggests that diabetes prevention strategies targeting high-risk individuals may require some degree of intervention maintenance.

**Added value of this study**

Our five-year post-randomisation follow-up study shows that PLA effects on knowledge and awareness of diabetes remain but mHealth effects are no longer observed. Positive impacts of PLA on diabetes and intermediate hyperglycaemia outcomes are no longer seen. However, measures of hypertension, hypertension control and exploratory analyses of key risk behaviours and additional measures of blood pressure suggest lasting positive health impacts of PLA.

**Implications of all the available evidence**

Population-level interventions that seek to address broad cultural and societal influences of cardiometabolic risk may require a strong focus on maintenance of interventions strategies and effect. Though impacts of our PLA community mobilisation intervention on blood glucose are no longer observable 5-years post-randomisation, whole-population, community-based awareness and lifestyle interventions that prevent the onset of diabetes, even if only temporarily, may cumulate and contribute to wider positive impacts on health, including health behaviours and blood pressure, and should remain a priority for populations with a high burden of risk.

## Introduction

Diabetes is a priority non-communicable disease (NCD) listed in the UN and WHO Action Plan to address the global burden of NCDs.^1^ The International Diabetes Federation estimate that approximately 700 million adults (10.9%) will live with diabetes by 2045 and the greatest burden of disease will be in low- and middle-income countries.^2^ Currently, around 79% of people with diabetes live in low- or middle-income countries, and more than 60% live in Asian countries. The estimated prevalence of diabetes in Bangladesh is 2–13%, depending on study design, methods and location, and the estimated prevalence of intermediate hyperglycaemia (impaired fasting glucose or impaired glucose tolerance) is between 2 and 22%.^3^ Of those living with diabetes in Bangladesh, it is estimated that 50–75% are undiagnosed and unaware of their condition.^4^^,^^5^ There is a need for effective and sustainable population-level interventions to raise awareness of and to prevent and control diabetes in settings such as Bangladesh, and a need for longitudinal evidence on the impact of these population-level interventions on diabetes and associated cardiometabolic risk.^6^

The DMagic (Diabetes Mellitus Action through Groups or Information for better Control) cluster randomised controlled trial showed that, after 18 months of a participatory learning and action (PLA) community mobilisation intervention, community awareness and understanding of type-2 diabetes mellitus (T2DM) was greatly increased and the odds of T2DM and intermediate hyperglycaemia was 64% lower in intervention villages than control villages (adjusted odds ratio (95% confidence interval) 0.36 (0.27, 0.48)).^7^ Further, among individuals identified with intermediate hyperglycaemia before the intervention, the cumulative two-year incidence of T2DM was 59% lower in intervention villages (0.41 (0.24, 0.67)). This equates with absolute reductions in prevalence and incidence of 21% for T2DM and 9% for intermediate hyperglycaemia. An mHealth intervention which was also tested in the DMagic trial, raised population knowledge and understanding of diabetes but had no effect on blood glucose measures when compared to controls.^7^ No intervention effects on BMI or other major risk factors for diabetes were observed in the DMagic trial.

The DMagic trial ended in 2018, and was the first evidence of population-level community-based interventions for diabetes prevention and control using PLA. Though not directly comparable to our intervention or context, evidence from other settings suggests that diabetes prevention strategies targeting high-risk individuals can achieve reductions in diabetes incidence that last for several years post-intervention, but that often some degree of intervention maintenance is required.^8^, ^9^, ^10^ In the current study we aim to describe the medium-term sustainability of observed intervention effects in the absence of intervention maintenance and, given hyperglycaemia is a key modifiable risk factor for the development of cardiovascular diseases (CVDs), explore possible positive effects on measures of blood pressure three years after the end of all intervention activity.

## Methods

### Setting

This study took place in Faridpur District, south-central Bangladesh. Faridpur has a population of over 1.7 million people in an area of just over 2000 km^2^ and is situated on the banks of the Padma River. The district has a mainly agricultural economy, with the main crops being jute and rice. The population is mainly Bengali and almost 90% of the population in Faridpur are Muslim, with the remaining population largely Hindu. Administratively, Faridpur District is divided into nine upazillas. Four upazillas in Faridpur District were purposefully selected because they were accessible to the district headquarters of the Diabetic Association of Bangladesh (BADAS) in Faridpur Sadar: these are Boalmari, Saltha, Madhukhali and Nagarkanda. For each of these upazillas, the 2011 Bangladesh Census^11^ was used to select 96 villages with population size of between 750 and 2500 (total estimated population 125,000).

### DMagic interventions & trial design

DMagic was a three-arm, cluster-randomised trial of participatory community mobilisation, mHealth mobile phone voice messaging, and usual care (control) in 96 villages. Community mobilisation involved 18 monthly group meetings, led by salaried lay facilitators, applying a PLA cycle focused on diabetes prevention and control. 122 groups comprised of an average of 27 members each were established across 32 villages. Each group was open to all community members and progressed through a four phase PLA cycle of problem identification and prioritisation, strategy development, strategy implementation and evaluation. Facilitators were locally recruited men and women who each led up to nine male or female groups, respectively, and helped groups to plan and coordinate activities, including wider community meetings that involved sharing learnings and strategies with others in the local area. Group strategies varied between groups and depending on local priorities, but common approaches included awareness raising, group exercises (especially walking groups), and locally organised diabetes screening.^12^

The mHealth intervention involved free twice-weekly voice messages sent to individual's mobile phones across 32 villages over 14 months promoting awareness and behaviour change to reduce diabetes risk.^13^ Voice messages were developed based on formative research and behaviour change theory, as described previously.^14^ Anyone residing in any of the 32 mHealth villages with access to a mobile phone could opt-in to receive the intervention messages free of charge and recipients were encouraged via the messages to share the content of messages with family and friends.

Stratified 1:1:1 randomisation of the 96 villages allocated them to the mHealth intervention, the community mobilisation intervention, or control, with each upazilla constituting one stratum. Because of the nature of the interventions being tested, the intervention team and participants could not be masked to allocation. The DMagic trial is registered with the ISRCTN registry (ISRCTN41083256). The current follow-up study was not part of the original trial design.

### Follow-up sample

Using the household sampling frame developed for the DMagic endline survey in 2017/18, a new sample of 20 adults aged ≥30 years was randomly selected from each study village (total n = 1920) in 2021. This sample size is based on 80% power, with 5% significance, to detect a 30% reduction in the primary outcome of T2DM and intermediate hyperglycaemia between the 32 PLA and 32 control clusters, assuming a 40% prevalence in the control clusters, intracluster correlation coefficient (ICC) of 0.07 and 20% non-response. In addition, we purposively sampled known individuals identified with intermediate hyperglycaemia in the DMagic baseline survey conducted in 2016 who were also located in the DMagic endline survey in 2018 (n = 2099).

Full details of how study clusters were sampled has been previously published.^13^ To select the current study sample, 20 households with at least one eligible adult were selected using simple random sampling. At the next stage, a single eligible adult from each household was selected for inclusion in the survey using simple random sampling. Eligibility was based on permanent residence of at least the past 6 months in the study village. Pregnancy was an exclusion criteria due to potential for gestational diabetes and other pregnancy-related metabolic, physiologic and anthropometric effects that were beyond the scope of our interventions.

### Outcomes

#### Pre-specified

As in the DMagic trial, we had two primary outcomes. 1) The combined prevalence of intermediate hyperglycaemia and T2DM among adults aged ≥30 years. This uses the same definition as in the DMagic trial and is based on WHO definitions and blood glucose cut-offs for normoglycaemia, impaired fasting glucose, impaired glucose tolerance and T2DM, or a prior diagnosis of T2DM by a medical professional^15^ (supplementary Table S1). 2) The five-year cumulative incidence of T2DM (defined according to WHO criteria or based on reported medical diagnosis of T2DM) among individuals identified with intermediate hyperglycaemia in the 2016 DMagic baseline (pre-intervention) survey.

Pre-specified secondary outcome measures were assessed among the random population sample. These were objective physical assessments of systolic and diastolic blood pressure, hypertension, hypertension control, mean BMI, proportion of overweight or obesity, and abdominal obesity (supplementary Table S1). In addition, we included survey-assessed measures of self-rated health (on scale of 0–100), diabetes knowledge (relating to causes, symptoms, complications, prevention and control) and proportion reporting a minimum of 150 min of physical activity per week. Among individuals reporting a prior diagnosis of diabetes we report diabetic control (defined as blood glucose levels below the diabetic threshold among individuals with a self-reported medical diagnosis of diabetes), self-reported receipt of medical diabetes treatment or advice, self-reported monthly blood glucose monitoring, and self-reported diabetes co-morbidities that respondents had been told by a medical professional were associated with diabetes.

Measures of psychological distress using the Self-Rated Health Questionnaire (SRQ-20) and the mean daily number of fruit and vegetable portions consumed were reported in our DMagic trial but were dropped from our 2021 survey.

#### Exploratory

In addition to the aforementioned outcomes that allow direct comparison with the DMagic trial analysis, we included outcomes that explore possible mechanisms or effects of the DMagic interventions. The selection of exploratory outcomes was based on findings from our process evaluation^12^ and visual participatory analysis^16^ of DMagic which indicated certain behaviours, practices and attitudes that had not been pre-specified in our trial analysis but were considered to be particularly important by intervention participants and could plausibly mediate intervention effects. These were: participation in brisk walking activities and time spent engaging in brisk walking, self-reported sugar consumption, salt consumption and oil consumption, 24 hour dietary diversity, depression and anxiety, and median score on the Appraisal of Diabetes Scale (ADS),^17^ a standardised diabetes-specific tool to evaluate a person's thoughts about coping with diabetes.^18^

We introduced new measures of depression using the Patient Health Questionnaire 9 (PHQ-9), which is a nine-item questionnaire designed to screen for depression and has been use previously and validated in Bangladesh.^19^, ^20^, ^21^ All participants were screened using the two-item PHQ-2 tool and those who screened positive for possible depressive disorder (a score of 3 or more) completed the full PHQ-9 survey. Anxiety was assessed using the Generalised Anxiety Disorder Assessment (GAD-7), a scale developed to identify probable cases of generalised anxiety and to assess symptom severity, which has previously been validated in Bangladesh.^22^^,^^23^

Considering that vascular biology and epidemiological evidence suggests that better-controlled blood glucose or delayed diabetes may confer cardiovascular disease benefit^24^ we explored PLA intervention impacts on additional measures known to be independently associated with an increased risk of stroke and ischaemic heart disease.^25^ These were: isolated systolic blood pressure, isolated diastolic blood pressure, and pulse pressure, which is an indicator of large blood vessel stiffness (supplementary Table S1).

### Procedures

Recruitment and training of data collectors took place in July 2021 and data collection took place between August–September 2021.

Sampled individuals were visited at their household, informed of the study and consent was obtained. All sampled individuals in a single cluster were informed of the anthropometric, blood glucose, and blood pressure measurement requirements of the study and were requested to attend a local centre on the morning of a specified day following an overnight fast. The centre was established by the field team for the purposes of the study and was at a central, convenient location in the village. Collection of questionnaire data took place at a private outside location near the respondent's home before or after the physical measurements or at the time of physical measurement in the testing centre. Data were linked using a study ID number.

Data were collected by 12 teams of fieldworkers comprised of a total of 28 men and women with at least secondary education who were recruited locally and selected through a written assessment and interview. All fieldworkers underwent 10 days training on survey methods and how to take physical measurements followed by one week supervised field practice and daily debriefs in villages in Faridpur that were not included in the study. Data collectors were supervised by four field supervisors with experience in survey methods. Each supervisor was responsible for three data collection teams, spending half a day observing and verifying data within each team at least every two days. Within each village, teams were aided by a village assistant, usually a young male, who received a daily payment to coordinate study participants and assist data collectors in their duties. Questionnaire data were gathered using Samsung Galaxy Grand Prime large screen smartphones using ODK Collect. All survey procedures were conducted in line with COVID-19 safety precautions, including the use of face masks, and were in line with Government of Bangladesh guidance at the time.

Detailed information on the sociodemographic characteristics of all sampled individuals were collected using a structured survey instrument adapted from the WHO Stepwise tool^26^ and the 2014 Bangladesh Demographic and Health Survey.^27^ This was designed to measure the background demographic and socio-economic characteristics, lifestyle and behavioural risk factors, diabetes awareness indicators and health seeking behaviour and costs of care seeking among study participants.

Fieldworkers measured blood pressure, blood glucose concentration, body weight, height, and waist and hip girth using standard methods. Blood pressure was measured using the OMRON HBP 1100 Professional Blood Pressure Monitor (Kyoto, Japan). Two measurements were taken at approximately 5-min intervals and the respondent's blood pressure obtained by averaging these measurements. Measurements of height, weight, and waist and hip girth were taken with light clothes without shoes. The weighing tools were calibrated daily by known weight. For height, the subject stood in erect posture vertically touching the occiput, back, hip, and heels on the wall while gazing horizontally in front and keeping the tragus and lateral orbital margin in the same horizontal plane. Waist girth was measured by placing a plastic tape horizontally midway between 12th rib and iliac crest on the mid-axillary line. Similarly, hip circumference was measured by taking the extreme end posteriorly and the symphysis pubis anteriorly.

Blood glucose was measured using the One Touch Varioflex Glucometer (Lifescan, Inc., Milpitas, CA 95035) in whole blood obtained by finger prick from capillaries in the middle or ring finger after an over-night fast. All individuals then received a 75 g glucose load dissolved in approximately 250 ml of water and had a repeat capillary blood test within 5 min of 120 min post ingestion to determine glucose tolerance status and differentiate between individuals with intermediate hyperglycaemia and those with diabetes according to WHO criteria.^15^ Individuals who reported a prior medical diagnosis of diabetes were not required to provide fasting and 2-h blood glucose measures but instead provided a random blood glucose sample. Although capillary blood glucose concentrations may overestimate blood glucose concentrations compared to venous samples, the method is feasible and acceptable for epidemiological studies and any measurement inaccuracy would be consistent across study arms.

Data were transferred from each data collectors’ tablet onto a laptop in the field every two days, by one of the field supervisors and gathered data were transferred from the laptop to the data manager in Dhaka once per week. Detected errors or requests for verification were sent back to the field team in Faridpur.

### Analysis

We compared the prevalence of intermediate hyperglycaemia and T2DM between clusters allocated to PLA, mHealth and control arm in the DMagic trial. All analysis was by intention-to-treat at the individual and cluster level, adjusting for clustering where appropriate and wealth quintile derived from principal components analysis (as in the DMagic trial). The intention-to-treat population only includes non-pregnant adults aged ≥30 years who are permanently residing in the village in which they were surveyed. Participants with missing data on the primary outcomes were excluded from primary outcome analysis, in-line with the primary analysis in the DMagic trial. Estimates of the intervention effects are presented with 95% confidence intervals. Analysis of primary outcomes was conducted by EF, who was blinded to intervention allocation, and results were shared with an independent trial steering committee before revealing allocation and proceeding with secondary outcome analyses.

Prespecified secondary outcomes and explanatory analyses were based on complete data only, i.e. cases with missing data on any outcome were excluded from that analysis. Comparative analysis used random-effects logistic regression for binary outcomes and mixed-effects linear regression for continuous outcomes, each allowing for clustering and upazilla stratification. Continuous outcome measures with a skewed distribution were log-transformed before regression analysis. Given that distinctive types of hypertension are strongly age-, sex- and BMI-dependent and correlate with hyperglycaemia,^28^^,^^29^ our exploratory analysis of isolated diastolic, isolated systolic and PP were also adjusted for age, sex, BMI and diabetic status.

No adjustments were made for the multiple statistical comparisons in this study on the basis that comparisons between trial arms and almost all outcomes replicate our a priori analysis plan for the DMagic trial and reflect the experimental design of the study. The explanatory outcomes, though not pre-specified as part of DMagic, were nonetheless defined as relevant outcomes based on process evaluation findings and prior to analysis of the 2021 data. All conducted comparisons are reported in this paper and our interpretation of results emphasises effect size, confidence intervals and consistency in intervention effects rather than focusing on p-values and arbitrary cut-off values of statistical significance.

All analyses were done using STATA/SE version 15.1.

#### Sensitivity

In view of the clinical relevance of T2DM as an outcome in its own right (i.e., not combined with intermediate hyperglycaemia), we did a post-hoc analysis in which we assessed intervention effects on a diabetes only outcome.

### Ethics

Written informed consent was obtained from all participants before data collection, or a thumb print for those unable to write. Ethical approvals for the DMagic trial and for this follow-up study were given by the University College London Research Ethics Committee (ref: 4766/002 and ref: 4199/007) and the Ethical Review Committee of the Diabetic Association of Bangladesh (ref: BADAS-ERC/EC/t5100246 and ref: BADAS-ERC/E/19/00276).

### Role of the funding source

The funder of the study had no role in study design, data collection, analysis, interpretation or writing of this paper. The corresponding author had full access to all the data in the study and had final responsibility for the decision to submit for publication.

## Results

### Response rates

**Fig. 1:**
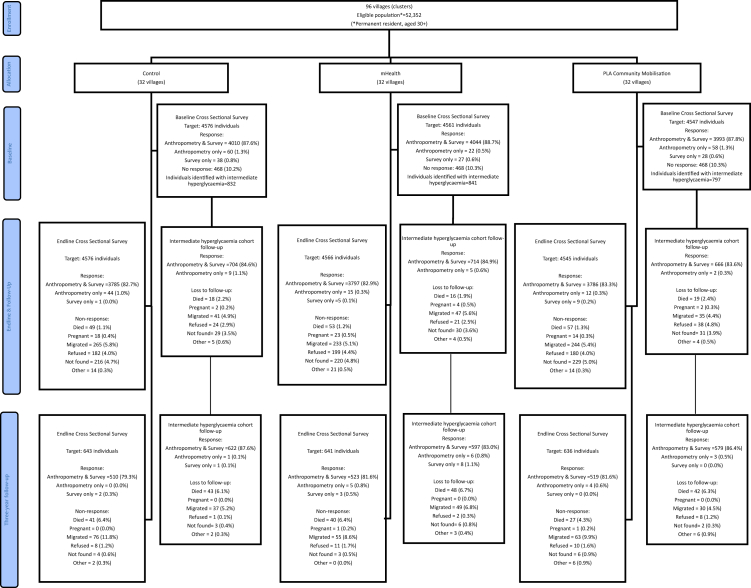
DMagic trial & follow-up profile.

Survey and/or anthropometric data were gathered from 1566/1920 (82%) of the random population sample (Fig. 1). The cross-sectional sample was similar in terms of sociodemographic characteristics between trial arms (Table 1). Non-responders were more likely to be men (213 (23%) of 936 men vs 141 (14%) of 984 women) and a similar pattern was observed across all arms. Reasons for non-response included migration (194 (54.8%)), death (108 (30.7%)), inability to locate (13 (3.7%)), or illness preventing participation (8 (2.3%)). Only 29 (8.2%) of the randomly sampled individuals refused to participate in the study. Reasons for non-response were generally similar across study arms, although death as a reason for non-response was higher in the control arm (31.3% (n = 41)) and mHealth arm (36.4% (n = 40)) compared to the PLA arm (23.9% (n = 27)).

**Table 1:** Sociodemographic characteristics among random sample between trial arms in 2021.

Sociodemographic parameter		Control	mHealth	PLA
*Cluster level (based on 2017 data)*				
Villages (Clusters)		32	32	32
Average village population aged ≥30 years (sd)		521 (189)	551 (152)	548 (225)
Average number of households (sd)		269 (97)	282 (79)	285 (112)
*Individual level*^a^				
Age	30–39 years 40–49 years 50–59 years 60–69 years 70–100 years	65 (12.7%) 194 (37.9%) 127 (24.8%) 75 (14.7%) 51 (10.0%)	64 (12.6%) 188 (35.4%) 134 (25.2%) 85 (16.0%) 55 (10.4%)	66 (12.6%) 184 (35.2%) 132 (25.2%) 81 (15.5%) 56 (10.7%)
Sex	Male Female	238 (46.5%) 274 (53.5%)	225 (42.4%) 301 (56.7%)	260 (49.7%) 259 (49.5%)
Education	None Primary Secondary Tertiary	231 (45.1%) 130 (25.4%) 149 (29.1%) 2 (0.4%)	238 (44.8%) 112 (21.1%) 171 (32.2%) 5 (0.9%)	239 (45.7%) 124 (23.7%) 155 (29.6%) 1 (0.2%)
Illiterate	Literate Illiterate	218 (42.6%) 294 (57.4%)	234 (44.1%) 292 (55.0%)	221 (42.3%) 298 (57.0%)
Marital status	Not married^b^ Married	75 (14.7%) 437 (85.4%)	95 (17.9%) 431 (81.2%)	61 (11.7%) 437 (85.4%)
Religion	Other Muslim	40 (7.8%) 472 (92.2%)	53 (10.0%) 473 (88.1%)	50 (9.6%) 469 (89.7%)
Respondent Occupation	No paid work Manual labour/trade Non-manual labour	303 (57.1%) 167 (31.5%) 61 (11.5%)	309 (58.2%) 171 (32.2%) 46 (8.7%)	273 (52.2%) 168 (32.1%) 78 (14.9%)
Wealth quintile	Most poor Very poor Poor Less poor Least poor	110 (21.5%) 95 (18.6%) 101 (19.7%) 114 (22.3%) 92 (18.0%)	122 (23.0%) 104 (19.6%) 115 (21.7%) 97 (18.3%) 88 (16.6%)	114 (21.8%) 91 (17.4%) 114 (21.8%) 85 (16.3%) 115 (22.0%)

a Data missing for all parameters for 9 respondents (0.6%) (5 individuals in mHealth arm and 4 individuals in the PLA arm) who participated in the physical and anthropometric measurements but not the interview survey.

b Including never married, widowed, separated & divorced.

Among the intermediate hyperglycaemia cohort of 2099 individuals, 1817 (87%) participated in the 2021 interview survey and/or anthropometric measurement (Fig. 1). Individuals lost to follow-up were more likely to be men (136 (18%) of 752 men vs 146 (11%) of 1347 women) and leading reasons for loss to follow-up were death (133 (47.2%)), migration (116 (41.1%)) and inability to locate (11 (3.9%)). Only 11 individuals (3.9%) refused to participate. A similar pattern of loss to follow-up was observed across all arms.

### Primary outcomes

No difference in the combined prevalence of T2DM and intermediate hyperglycaemia or the 5-year cumulative incidence of T2DM among the intermediate hyperglycaemia cohort was observed between intervention arms relative to control (Table 2).

**Table 2:** 2021 frequency, proportions and relative (odds ratio) and absolute (coefficient) effects and 95% confidence interval comparing normoglycaemia and intermediate hyperglycaemia and diabetes according to WHO diagnostic criteria21 a) among the random survey population (outcome 1), and b) among the intermediate hyperglycaemia cohort (outcome 2). Results are adjusted for (i) the stratified, clustered design, and (ii) the stratified, clustered design and adjustment for household wealth quintile.

Outcome 1: Population prevalence of intermediate hyperglycaemia and diabetes			
**Glycaemic status**	**Control**	**mHealth**	**PLA**
Normoglycaemic	309 (60.6%)	312 (59.1%)	291 (55.6%)
Diabetic or intermediate hyperglycaemic	201 (39.4%)	216 (40.9%)	232 (44.4%)
Total	510 (100.0%)	528 (100.0%)	523 (100.0%)
**Relative difference odds ratio (95% CI)**	**Control**	**mHealth**	**PLA**
(i) adjusted for stratified, clustered design	Reference	1.07 (0.77, 1.48); p = 0.69	1.23 (0.89, 1.70); p = 0.21
(ii) adjusted for (i) plus wealth	Reference	1.08 (0.78, 1.51); p = 0.64	1.23 (0.87, 1.74); p = 0.24
**Absolute risk difference (95% CI)**	**Control**	**mHealth**	**PLA**
(i) adjusted for stratified, clustered design	Reference	1.49 (−6.35, 9.33); p = 0.71	4.80 (−3.13, 12.7); p = 0.24
(ii) adjusted for (i) plus wealth	Reference	1.77 (−5.78, 9.33); p = 0.65	4.76 (−3.25, 12.8); p = 0.24
**Outcome 2: Five-year cumulative incidence among intermediate hyperglycaemic cohort**			

**Glycaemic status**	**Control**	**mHealth**	**PLA**
Normoglycaemic	272 (43.7%)	252 (41.8%)	263 (45.2%)
Intermediate hyperglycaemic	237 (38.0%)	232 (35.7%)	208 (35.7%)
Diabetic	114 (18.3%)	119 (19.7%)	111 (19.1%)
Total	623 (100.0%)	603 (100.0%)	582 (100.0%)
**Relative difference odds ratio (95% CI)**	**Control**	**mHealth**	**PLA**
(i) adjusted for stratified, clustered design	Reference	1.07 (0.77, 1.48); p = 0.69	1.04 (0.78, 1.40); p = 0.77
(ii) adjusted for (i) plus wealth	Reference	1.09 (0.78, 1.52); p = 0.61	1.04 (0.77, 1.41); p = 0.81
**Absolute risk difference (95% CI)**	**Control**	**mHealth**	**PLA**
(i) adjusted for stratified, clustered design	Reference	0.95 (−4.18, 6.08); p = 0.72	0.54 (−4.34, 5.42); p = 0.83
(ii) adjusted for (i) plus wealth	Reference	1.22 (−4.05, 6.50); p = 0.65	0.40 (−4.62, 5.42); p = 0.88

### Secondary outcomes

The prevalence of hypertension was lower in PLA clusters relative to control (adjusted odds ratio (aOR) (95% confidence interval): 0.73 (0.54, 0.97), p = 0.031) and individuals with a diagnosis of hypertension in PLA clusters were more than twice as likely to have controlled blood pressure relative to control clusters (aOR (95%CI): 2.77 (1.34, 5.75), p = 0.0061) (Table 3). There was no evidence of an effect of either intervention on measures of overweight and obesity, self-rated health, or proportion of respondents participating in at least 150 min of physical activity per week (Table 3). Although knowledge and understanding of diabetes in terms of its causes, symptoms, complications, prevention and control was generally high in all arms, it was higher in PLA villages compared to control and improvements in knowledge observed in the mHealth arm at the end of the DMagic trial were no longer statistically different to control.

**Table 3:** 2021 frequency, proportions and relative (odds ratio) and absolute (coefficient) effects and 95% confidence interval comparing pre-specified secondary outcomes adjusted for (1) the stratified, clustered design, and (2) the stratified, clustered design and adjustment for household wealth quintile.

Outcomes		Allocation			Crude^1^		Adjusted^2^	
		PLA	mHealth	Control	PLA vs Control	mHealth vs Control	PLA vs Control	mHealth vs Control
**Objective physical measures**								
Blood pressure	Mean diastolic blood pressure (sd)	74.2 (10.2)	75.2 (10.9)	74.6 (11.7)	−0.36 (−2.24, 1.51); p = 0.71	0.47 (−1.34, 2.28); p = 0.61	−0.32 (−2.24, 1.59); p = 0.74	0.49 (−1.37, 2.35); p = 0.61
	Mean systolic blood pressure (sd)	121.4 (16.9)	122.9 (18.8)	123.2 (20.2)	−1.72 (−4.63, 1.19); p = 0.25	−0.32 (−3.07, 2.44); p = 0.82	−1.68 (−4.66, 1.30); p = 0.27	−0.16 (−3.00, 2.68); p = 0.91
	Hypertension (%)	109 (20.8%)	135 (25.6%)	136 (26.7%)	0.72 (0.54, 0.96); p = 0.026	0.94 (0.71, 1.24); p = 0.67	0.73 (0.54, 0.97); p = 0.031	0.96 (0.72, 1.26); p = 0.75
	Hypertension control (%)	41 (66.1%)	41 (47.7%)	34 (42.5%)	2.69 (1.33, 5.44); p = 0.0061	1.27 (0.68, 2.37); p = 0.46	2.77 (1.34, 5.75); p = 0.0061	1.31 (0.69, 2.51); p = 0.41
Overweight & obesity	Mean Body Mass Index (BMI) (sd)	22.4 (3.8)	22.3 (3.8)	22.4 (3.7)	0.02 (−0.45, 0.48); p = 0.95	−0.08 (−0.55, 0.39); p = 0.73	0.01 (−0.43, 0.45); p = 0.97	−0.01 (−0.45, 0.43); p = 0.97
	Overweight or obese (%)	214 (40.9%)	214 (40.5%)	209 (41.0%)	0.99 (0.76, 1.30); p = 0.96	0.99 (0.77, 1.26); p = 0.93	1.00 (0.77, 1.31); p = 0.98	1.03 (0.79, 1.34); p = 0.84
	Abdominal obesity (%)	163 (62.9%)	205 (68.1%)	193 (70.7%)	0.68 (0.44, 1.07); p = 0.097	0.86 (0.55, 1.35); p = 0.52	0.69 (0.43, 1.12); p = 0.14	0.90 (0.57, 1.43); p = 0.65
**Interview survey measures**								
Quality of life & wellbeing	Median Self Rated Health (IQR)	80 (70–95)	80 (70–95)	80 (70–90)	0.00 (−0.04, 0.04); p = 0.99	−0.03 (−0.08, 0.01); p = 0.14	0.00 (−0.04, 0.04); p = 0.99	−0.03 (−0.08, 0.01); p = 0.16
Diabetes knowledge	Ability to report one or more valid *causes* of diabetes (%)	459 (88.4%)	409 (77.8%)	382 (74.6%)	3.95 (1.39, 11.24); p = 0.010	1.14 (0.49, 2.62); p = 0.76	3.97 (1.38, 11.43); p = 0.011	1.17 (0.50, 2.70); p = 0.72
	Ability to report one or more valid *symptoms* of diabetes (%)	473 (91.1%)	440 (83.7%)	410 (80.0%)	4.43 (1.55, 12.62); p = 0.0054	1.27 (0.61, 2.62); p = 0.53	4.42 (1.55, 12.62); p = 0.0055	1.31 (0.63, 2.71); p = 0.47
	Ability to report one or more valid *complications* of diabetes (%)	447 (86.1%)	398 (75.7%)	364 (71.1%)	5.08 (1.63, 15.79); p = 0.0050	1.40 (0.59, 3.32); p = 0.45	5.19 (1.64, 16.35); p = 0.0049	1.42 (0.60, 3.39); p = 0.43
	Ability to report one or more valid ways to *prevent* diabetes (%)	472 (90.9%)	451 (85.7%)	416 (81.3%)	4.05 (1.45, 11.30); p = 0.0075	1.30 (0.66, 2.56); p = 0.45	4.02 (1.44, 11.22); p = 0.0080	1.34 (0.68, 2.62); p = 0.40
	Ability to report one or more valid ways to *control* diabetes (%)	475 (91.5%)	466 (88.6%)	439 (85.7%)	3.37 (1.28, 8.85); p = 0.014	1.24 (0.70, 2.19); p = 0.46	3.27 (1.23, 8.68); p = 0.017	1.27 (0.72, 2.23); p = 0.41
Physical Activity	Average of 150 min or more doing physical activity per week (%)	291 (56.1%)	258 (49.1%)	289 (56.5%)	0.99 (0.63, 1.56); p = 0.97	0.71 (0.45, 1.14); p = 0.16	0.99 (0.63, 1.56); p = 0.97	0.72 (0.45, 1.14); p = 0.16
**Among individuals with diabetes**								
Diabetes awareness & care	Self-awareness of diabetic status (%)^a^	39 (42.4%)	28 (37.3%)	26 (39.4%)	1.05 (0.54, 2.03); p = 0.90	0.86 (0.43, 1.74); p = 0.68	1.10 (0.55, 2.23); p = 0.79	0.85 (0.41, 1.73); p = 0.65
	Diabetes control (%) (random blood glucose<11.1 mmol/l)^b^	22 (56.4%)	21 (72.4%)	17 (65.4%)	0.71 (0.25, 2.01); p = 0.52	1.28 (0.38, 4.31); p = 0.69	0.53 (0.17, 1.72); p = 0.29	1.40 (0.32, 6.06); p = 0.65
	Receipt of professional treatment or advice for diabetes (%)^b^	37 (94.9%)	25 (89.3%)	23 (85.2%)	3.42 (0.56, 20.81); p = 0.18	1.89 (0.17, 21.62); p = 0.61	3.54 (0.54, 23.35); p = 0.19	2.18 (0.11, 42.74); p = 0.61
	Minimum monthly blood glucose testing (%)^b^^c^	19 (48.7%)	10 (35.7%)	11 (40.7%)	1.31 (0.46, 3.80); p = 0.61	0.83 (0.26, 2.61); p = 0.75	0.91 (0.28, 2.93); p = 0.88	0.68 (0.19, 2.41); p = 0.55
	Diabetes-related complications (%)^b^^d^	29 (74.4%)	21 (75.0%)	20 (74.1%)	0.76 (0.21, 2.67); p = 0.66	0.80 (0.07, 9.12); p = 0.86	0.35 (0.07, 1.70); p = 0.19	0.19 (0.00, 168.81); p = 0.63

a Among those identified as diabetic by objective blood glucose test (n = 233).

b Among individuals with self-reported diabetes (n = 94).

c Missing information for 3 individuals (2 mHealth; 1 control).

d Missing information on complications for 4 individuals (2 mHealth; 2 control).

Among the 233 individuals with blood glucose readings indicating T2DM, 93 (39.9%) reported a prior diagnosis of diabetes, with no difference in awareness between trial arms. Among a total of 94 individuals reporting a prior diagnosis of diabetes (1 had no blood glucose reading), approximately two thirds (n = 60, (63.8%)) had random blood glucose levels lower than 11.1 mmol/l, indicating diabetic control. The proportion of people with controlled diabetes was lower in the PLA arm (56.4%) compared to mHealth (72.4%) and control (65.4%), though reported receipt of professional treatment and advice, and at least monthly blood glucose monitoring was higher in the PLA arm. None of the observed numerical differences in diabetes care indicators were statistically significant.

### Exploratory outcomes

Exploratory analysis of behavioural and health outcomes indicated that whilst individuals living in PLA clusters were no more likely to participate in brisk walking activities compared to individuals in control clusters, they spent on average 31% (approximately 55 min) longer doing this activity per week (Table 4). No significant differences in dietary habits were observed and although mean ADS score was lower in the PLA arm compared to control (indicating more positive appraisal), this was not statistically significant. On average, individuals in the PLA arm scored lower on the PHQ-2 screening tool, however, of those who did screen positive and who completed the PHQ-9 tool, individuals in the PLA arm scored significantly higher than those in the control arm, indicating more severe depressive symptoms.

**Table 4:** 2021 frequency, proportions and relative (odds ratio) and absolute (coefficient) effects and 95% confidence interval comparing exploratory outcomes adjusted for (1) the stratified, clustered design, (2) the stratified, clustered design and adjustment for household wealth quintile, and (3) (hypertensive outcomes only) the stratified, clustered design and adjustment for household wealth quintile, age group, sex, diabetic status and BMI.

Outcomes				Crude^1^	Adjusted^2^	Adjusted^3^
		Community PLA	Control	PLA vs Control	PLA vs Control	PLA vs Control
Walking	Participates in brisk walking (%)	93 (17.9%)	79 (15.4%)	1.43 (0.80, 2.55); p = 0.23	1.40 (0.79, 2.46); p = 0.23	
	Median (IQR) time spent brisk walking per week	240 (180–360)	180 (120–300)	0.27 (0.04, 0.49); p = 0.019	0.27 (0.02, 0.51); p = 0.031	
Diet	Mean 24 Hour Dietary Diversity Score (DDS) (sd)	6.72 (1.86)	6.61 (1.93)	0.10 (−0.22, 0.42); p = 0.54	0.08 (−0.22, 0.38); p = 0.59	
	No added sugar to foods in previous 24 h	178 (39.6%)	154 (37.6%)	1.07 (0.78, 1.47); p = 0.65	1.08 (0.79, 1.48); p = 0.62	
	No added salt to foods	145 (27.9%)	125 (24.4%)	1.19 (0.86, 1.65); p = 0.31	1.17 (0.84, 1.64); p = 0.34	
	Mean (sd) monthly household oil consumption	4.62 (1.32)	4.74 (1.47)	−0.12 (−0.36, 0.11); p = 0.31	−0.13 (−0.36, 0.10); p = 0.25	
Appraisal of Diabetes Scale (ADS)	Mean (SD) ADS score among known diabetics (n = 97)	12.2 (3.37)	13.9 (4.93)	−1.39 (−3.53, 0.76); p = 0.21	−0.61 (−2.84, 1.62); p = 0.59	
Depression & anxiety	Median (IQR) PHQ2 score (depression screening)	1 (0–2)	1 (0–2)	−0.12 (−0.23, −0.01); p = 0.026	−0.12 (−0.22, −0.01); p = 0.028	
	Mean (SD) PHQ9 score (among PHQ2 screen positive, n = 142)	13.3 (4.8)	11.1 (4.6)	2.21 (0.08, 4.34); p = 0.042	2.27 (0.12, 4.43); p = 0.039	
	Median (IQR) GAD7 score (anxiety)	3 (1–6)	3 (1–6)	−0.07 (−0.23, 0.09); p = 0.41	−0.06 (−0.21, 0.09); p = 0.44	
Blood pressure measures	Isolated systolic hypertension^a^	37 (7.1%)	51 (10.0%)	0.69 (0.42, 1.16); p = 0.16	0.70 (0.42, 1.17); p = 0.18	0.62 (0.36, 1.06); p = 0.081
	Isolated diastolic hypertension^a^	7 (1.3%)	15 (2.9%)	0.44 (0.18, 1.10); p = 0.080	0.42 (0.17, 1.05); p = 0.063	0.41 (0.16, 1.04); p = 0.060
	Mean pulse pressure (sd)	47.2 (12.0)	48.6 (13.8)	−1.39 (−3.15, 0.38); p = 0.13	−1.38 (−3.16, 0.40); p = 0.13	−1.78 (−3.34, −0.22); p = 0.026

a Denominator is all non-cases.

Blood pressure-related measures of cardiovascular risk suggest a possible positive advantage in PLA clusters relative to control, particularly in terms of mean pulse pressure and with adjustment for known correlates of age, sex, BMI and diabetic status. There was no evidence of mHealth intervention effect on any of the exploratory outcomes (supplementary Table S2).

As per our DMagic analysis, and in view of the clinical relevance of T2DM as an outcome in its own right (i.e., not combined with intermediate hyperglycaemia), we did a post-hoc analysis in which we assessed intervention effects on a diabetes-only outcome. The adjusted odds of diabetes was 45% higher in PLA clusters compared to control (1.45 (1.03, 2.04); p = 0.034), and no significant effect was observed in mHealth clusters relative to control (1.13 (0.78, 1.64); p = 0.51).

## Discussion

Our five-year post-randomisation follow-up of the DMagic cluster randomised controlled trial shows that whilst knowledge about diabetes remains significantly higher in PLA clusters relative to control, intervention effects on blood glucose outcomes are no longer observed. Improvements in knowledge among the mHealth clusters that we measured in 2018 were also no longer observable in 2021.

The DMagic interventions did not specifically target high-risk individuals, but rather employed a broad population-level approach to prevention and control and our trial design similarly assessed population level outcomes among individuals residing in study clusters rather than just those directly exposed to and engaged with the interventions. We know from process evaluation that the PLA community mobilisation intervention stimulates change at the individual, household and community levels that enable, reinforce and amplify impacts, such that effects are observed even in those who do not directly engage with the intervention.^30^ It is therefore challenging to directly compare our findings to those from other targeted diabetes intervention follow-up studies and furthermore, there is a lack of such studies from LMICs. Nevertheless, there is evidence from high-income settings that lifestyle modification interventions among high-risk groups can be promising long-term diabetes prevention strategies. However, the need for some degree of maintenance intervention to observe prolonged effects is noted.^10^ All DMagic intervention activities ended in 2017 and there has been no further support or maintenance to PLA groups or mHealth since then. While there was a community hand-over process for groups, unpublished data from our surveys indicates that none of the 122 PLA groups established in DMagic met later than 2018, and we are not aware of any other population-based interventions for diabetes prevention and control in our study areas since DMagic. Previous follow-up of PLA for maternal and neonatal health suggests that groups may be sustainable^31^ and so more understanding is needed on what aspects of our study context, diabetes-focus and handover might have influenced sustainability of DMagic PLA groups.

Despite large impacts of PLA on the prevalence of diabetes and intermediate hyperglycaemia and the two-year incidence of diabetes among the intermediate hyperglycaemia cohort in 2018, these primary outcomes did not differ significantly between the three randomised groups in 2021. This finding differs from diabetes intervention follow-up studies in China^8^ and Finland,^9^ which showed that reduction in diabetes incidence remained for several years after the period of active intervention. However, these studies were in high-risk individuals. Similar to the Diabetes Prevention Program Outcome Study (DPPOS),^32^ our findings may be attributable to a fall in the incidence of diabetes and intermediate hyperglycaemia in the control and mHealth arms due to the majority of individuals susceptible to intermediate hyperglycaemia and diabetes developing these outcomes during the initial DMagic trial, leaving a reduced number at risk in subsequent years. Our data might also indicate a rebound effect, whereby our PLA intervention did not prevent intermediate hyperglycaemia and diabetes, but rather delayed the onset of hyperglycaemia in susceptible individuals. We do not have reliable data on the date of onset of intermediate hyperglycaemia or diabetes in our study but can assume a substantial increase in incidence in PLA clusters post-2018 to result in comparable five-year (2016–2021) incidence in all trial arms. Possible delayed diabetes and a potential survival effect of DMagic PLA interventions (i.e. those with later diabetes surviving longer) are also plausible explanations for observed primary outcomes and especially the higher prevalence of diabetes only outcomes in the PLA arm in the absence of observable changes in diabetes risk such as BMI or risk behaviours.

Evidence from the Da Qing Diabetes Prevention Outcome Study of lifestyle interventions among high-risk individuals in China indicates that a delay in diabetes onset is associated with fewer cardiovascular events, lower incidence of microvascular complications, fewer cardiovascular disease deaths, fewer all-cause deaths and an average increase of life expectancy.^33^ Hyperglycaemia is a key modifiable risk factor for CVD risk and so effective reduction of hyperglycaemia, even if temporary, may have a positive effect on CVD risk.^6^ Further, increased risk of CVD associated with diabetes is augmented with coexistent hypertension, thus lower blood pressure and controlled hypertension promotes vascular health and may be especially important in reducing microvascular and macrovascular complications of diabetes. We observed improvements in hypertension, hypertension control and pulse pressure in PLA clusters relative to control in our follow-up. The exploratory nature of our analysis of isolated pressures and pulse pressure and multiple hypothesis testing notwithstanding, the role of blood pressure lowering to improve prognosis in T2DM is well-established^24^^,^^29^^,^^34^ and these observations are potentially important if they translate to lower cardiovascular risk in PLA communities.

Existing population attributable risk estimates suggest that even small decreases in population blood pressure can result in large decreases to overall cardiovascular risk in the population.^35^ Though observed absolute differences in mean diastolic and systoloic blood pressure in our study are relatively small between study arms, the lower mean and smaller standard deviation of blood pressure measures in the PLA arm compared to control infer a significantly decreased probability that individuals in the PLA arm will reach the diastolic or systolic thresholds for hypertension. Indeed, our observed relative reduction of odds of hypertension by 27% and more than two-fold increase in control of blood pressure among individuals with hypertension associated with PLA clusters suggest positive and plausible lasting impacts of the PLA intervention on blood pressure. Further, our observed, though not statistically significant, lower odds of isolated blood pressures and reduction of −1.78 mmHg pulse pressure (when adjusted for age, sex, wealth, BMI and diabetes status) could convey meaningful reductions in population risk of ischaemic heart disease and stroke.^36^

Though our DMagic interventions did not specifically target blood pressure, raised blood pressure and raised blood glucose share several common risk factors and so many of the possible PLA intervention mechanisms that reduce diabetes risk could plausibly also have beneficial effects on blood pressure. However, despite the large observable effects on blood glucose in 2018, intervention effects on hypertension^7^ and measures of blood pressure (retrospective analysis in supplementary Table S3) were not observed in our 2018 data. There may be several reasons for this, including a possible inertia in blood pressure that means that changes resulting from intervention mechanisms take longer to have an effect. Alternatively (or in addition), the observed improvements in blood pressure might themselves be mediated by improvements in blood glucose and so may not be expected to occur simultaneously with reductions in intermediate hyperglycaemia and diabetes.

We do not have measures of the quantity of salt consumed by study participants or the relative or absolute changes in the amount of salt consumed, but small reductions in salt being added to food were observed in PLA clusters relative to control. Reduction of salt consumption is one of the most effective ways to reduce blood pressure in populations and individuals and may have contributed to the changes in blood pressure we observed.^37^ Effective reduction of population salt consumption is likely to require a combination of targeted salt reduction strategies as well as community interventions – such as PLA – that address individual and contextual factors influencing dietary salt use.

The Framingham Heart Study reported that, with increasing age, a shift from diastolic to systolic hypertension and then to pulse pressure was a predictor of coronary heart disease.^38^ Isolated diastolic hypertension is a relatively uncommon hypertension phenotype but is associated with increased stroke, heart disease and other sequalae of hypertension.^25^ Pulse pressure is a marker for increased large arterial stiffness and is a major independent predictor of cardiovascular mortality and atrial fibrillation^39^ and as little as 10 mmHg increase in pulse pressure can increase cardiovascular risk by approximately 20%.^40^ Although mean pulse pressure observed in our study is within a normal range in PLA and control clusters and the epidemiological and clinical significance of the small observed difference is uncertain, further follow-up would be valuable.

As in the original DMagic trial analysis, there are no major differences in behavioural outcomes. The overall number of individuals living with diabetes who were aware of their status was higher than in 2018, but still low and so comparisons of diabetes-specific behaviours lack statistical power. Nevertheless, taken together, the greater knowledge of diabetes symptoms, prevention and control in PLA clusters and numerically (though not statistically significant) higher levels of awareness, receipt of professional treatment/advice and regular blood glucose monitoring, albeit with lower levels of control, and the lower (more positive) ADS score suggest there may be some differences in how people live and experience diabetes in PLA clusters compared to control and mHealth clusters. The fact that, despite higher prevalence of diabetes in PLA clusters relative to control, measures of self-rated health and self-reported complications of diabetes did not differ between arms may further indicate either more recent progression to diabetes or better self-management in PLA clusters, although our measure of diabetes control contradicts the latter.

The proportion of the population engaging in an average of at least 150 min of physical activity per week was lower in our 2021 survey compared to 2018, with no difference between study arms. The reason for this decline in physical activity is unknown but may plausible be related to a global decline in physical activity associated with the COVID-19 pandemic and imposed restrictions, as observed in other South Asian settings.^41^^,^^42^ Walking was identified as a critical strategy of the PLA intervention in DMagic, with the intervention addressing socio-cultural barriers to walking for exercise.^12^^,^^43^ We therefore conducted exploratory analysis of brisk walking as an activity and observed that, although the proportion of people engaging in this activity did not differ between PLA and control arms, the time spent brisk walking was almost 1 hour longer per week in PLA villages. Physical activity is known to have favourable effects on cardiometabolic health and, though the optimal frequency and intensity of physical activity is not universally defined, brisk walking has been identified as an appropriate and accessible form a physical activity that can be practiced by individuals and groups without cost and with low risk of injury.^44^

With the possible exception of time spent engaged in brisk walking, there was no evidence of mHealth intervention effect on any of the exploratory outcomes (supplementary Table S2). This is perhaps not surprising given that primary and secondary outcomes did not change in the mHealth arm in DMagic. The possible effect on walking time may be spurious given it is inconsistent with the absence of other mHealth intervention effects outcomes.

A limitation of our study is that our sample size, though large, was designed to assess primary outcomes and several of our analyses of secondary and exploratory outcomes lack statistical power. Like many diabetes intervention studies, our assessment is relatively short-term, lacks intermediary measures of outcomes since the end of intervention and focuses on blood sugar, self-reported behaviours and relatively short-term cardiometabolic risk markers. Nevertheless, high response and follow-up rates, rigorous field methods, including fasting and 75 g oral glucose tolerance tests among a representative population-based sample of people in rural Faridpur are strengths of our study. Further, the robust design of the original DMagic trial, including cluster randomisation of 96 villages with limited contamination between clusters and the intention-to-treat analysis of this follow-up observational study enhance validity of our findings. Finally, it is important to note we did not apply any statistical corrections for multiple hypothesis testing in our analysis on the basis that the comparisons were by in lagre pre-specified and part of our original experimental design. Nevertheless, interpretation of results should focus on effect size and confidence intervals and consistency of intervention effects across outcomes rather than on concepts of absolute statistical significance.

Adult metabolic health is a complex interaction of blood glucose, blood pressure, obesity, sex and age. Delayed hyperglycaemia and small changes in physical activity, dietary and diabetes-related behaviours associated with exposure to DMagic PLA community mobilisation intervention may cumulate and contribute to positive impacts on blood pressure, an important markers of cardiovascular risk, five years after randomisation. By addressing broad cultural and societal influences of cardiometabolic risk, whole-population, community-based awareness and lifestyle interventions are likely to be cost-effective strategies to reach large groups of people with potential to affect the entire distribution of disease risk, even if only by a small degree, to affect the proportion of those at risk.^6^ It is likely that sustained changes in social norms and associated benefits of these require a strong focus on maintenance, which in the context of DMagic, could include strategies for intermittent follow-up, incentivisation to groups and remote support, including using digital health technologies. Interventions that prevent the onset of diabetes, even if only temporarily, should remain a priority for populations with a high burden of risk since even short-term delay may postpone diabetes related complications and costly care. Longer-term follow-up are needed to fully understand lasting intervention effects on diabetes onset, cardiovascular complications and mortality.

## Contributors

EF, project Principal Investigator, led in the design of the study, conducted statistical analyses and drafted the manuscript. CK provided technical coordination of the survey and data management process and contributed to analysis and interpretation. NA contributed to intervention development, survey methods and interpretation. SKS coordinated quantitative data collection activities, data management and interpretation. JM led the process evaluation component of the study and contributed to intervention development and interpretation of findings. MP contributed to survey design and interpretation. AK contributed to project management, survey and intervention coordination and interpretation of study findings. TN developed and coordinated the implementation of PLA group activities. HHB contributed to survey design and interpretation. AKAK provided technical oversight of all project activities and facilitated community engagement and intervention development activities. KA coordinated project activities in Bangladesh, co-led the project and contributed to intervention development and study design. All authors have reviewed and contributed to the reporting of study findings in this paper.

## Data sharing statement

De-identified data collected for this study and a data dictionary are available from the corresponding author on reasonable request.

## Declaration of interests

Recipients of funding for this work were Principal Investigator Fottrell and Co-investigators Azad, Khan, Kuddus, Haghparast-Bidgoli, Morrison and King. Co-authors Ahmed, Shaha, Pires and Nahar were employed on the project using project funding.

The authors declare no competing interests.

## Appendix A Supplementary data

Abstract Bangla
