# Supplementary material for: Intermediate hyperglycaemia, diabetes and blood pressure in rural Bangladesh: five-year post-randomisation follow-up of the DMagic cluster-randomised controlled trial
Source: Lancet Reg Health Southeast Asia. 2022 Dec 10;10:100122. doi: 10.1016/j.lansea.2022.100122 (PMC10015271; doi:10.1016/j.lansea.2022.100122)
Supplement: Abstract Bangla [file mmc1.pdf]

Disclaimer:This translation in [language] was submitted by the authors and we reproduce it as supplied. It has not been peer reviewed. Our editorial processes have only been applied to the original abstract in English, which should serve as reference for this manuscript

Translated abstract

ইন্টারমেডিয়েট হাইপারগ্লাইসেমিয়া, ডায়াবেটিস এন্ড ব্লাডপ্রেসার ইন রুরাল বাংলাদেশ: ফাইভ ইয়ার পোষ্ট-রেন্ডমাইজেশান ফলোআপ অব দ্য ডি-ম্যাজিক ক্লাষ্টার-রেন্ডমাইজড ট্রায়াল

এডওয়ার্ড ফট্রেল[১]*, কেরিনা কিং[২], নাভিদ আহমেদ[৩], সঞ্জীত কুমার সাহা[৩], জোয়ানা মরিসন[১], মালিনী পিয়ার্স[১], আব্দুল কুদ্দুছ[৩], তাসমিন নাহার[৩], হাসান হাগপারেষ্ট বিদগলি[১], এ কে আজাদ খান[৩], কিশোয়ার আজাদ[৩]

১. ইউসিএল ইন্সষ্টিটিউট ফর গ্লোবাল হেলথ, ইউনিভার্সিটি কলেজ লন্ডন, ইউকে

২. ডিপার্টমেন্ট অব গ্লোবাল হেলথ, কেরোলিনস্কা ইন্সষ্টিটিউট, ষ্টকহোম, সুইডেন

৩. সেন্টার ফর হেলথ রিসার্চ এন্ড ইমপ্লিমেন্টেশন, ডায়াবেটিক এসোসিয়েশন অব বাংলাদেশ, ঢাকা, বাংলাদেশ

করেসপন্ডেন্স টু: ই. ফট্রেল, ইউসিএল ইন্সষ্টিটিউট ফর গ্লোবাল হেলথ, ৩০ গিলফোর্ড ষ্ট্রীট, লন্ডন, ডাব্লিউ ও সি ওয়ান এন ওয়ান ই এইচ, ইউকে; e.fottrell@ucl.ac.uk

পটভূমি
ইতিপূর্বে দ্য ডি-ম্যাজিক ট্রায়ালের ফলাফল হিসেবে দেখা গিয়েছিল যে, অংশগ্রহণমূলক শিক্ষা ও কর্মের দ্বারা কমিউনিটিকে কার্যকরী করার কমিউনিটি গ্রুপ এবং মোবাইল ফোনের কণ্ঠস্বর বার্তা (ভয়েস এস এম এস) প্রেরণের মাধ্যমে বাংলাদেশের ফরিদপুর জেলার গ্রামাঞ্চলের নির্দিষ্ট জনগোষ্ঠীর মধ্যে ডায়াবেটিস সম্পর্কে জ্ঞান বৃদ্ধি পেয়েছে এবং অংশগ্রহণমূলক শিক্ষা ও কর্মের প্রয়োগের ফলে ডায়াবেটিস হওয়ার ঘটনা হ্রাস পেয়েছে। প্রকল্পের কার্যক্রম শেষ হওয়ার তিন বছর পর আমরা উক্ত কার্যক্রমের ফলাফল মূল্যায়ন করেছি।

পদ্ধতি
২০১৬ সালে অনির্দিষ্ট বাছাইকরনের মাধ্যমে যে ৯৬ টি গ্রামে ডি-ম্যাজিকের কাজ শুরু হয়েছিল, পাঁচ বছর পর সেই গ্রামগুলো থেকে অনির্দিষ্ট বাছাইকরণের মাধ্যমে ত্রিশ বছর বা তার বেশী বয়সের নারী'পুরুষের মধ্যে ক্রস সেকশনাল জরিপ পরিচালনা করা হয়েছিল এবং যাদের ইন্টারমিডিয়েট হাইপারগ্লাইসিমিয়া আছে তাদেরকে একটা দলে অন্তর্ভুক্ত করা হয়। প্রাথমিক ফলাফলগুলো ছিল:
১) ইন্টারমিডিয়েট হাইপারগ্লাইসিমিয়া এবং ডায়াবেটিসের সম্মিলিত প্রাদুর্ভাব;
২) ২০১৬ সালে নির্দিষ্ট করা অন্তর্বর্তী হাইপারগ্লাইসিমিয়া দলের মধ্যে পাঁচ বছরের সম্মিলিত ডায়াবেটিসের ঘটনা।
মাধ্যমিক ফলাফলগুলো ছিল: ওজন-উচ্চতা, বিএমআই, কোমড় এবং নিতম্বের পরিধির পরিমাপ, রক্তচাপ পরিমাপ, জ্ঞান ও আচরণসমূহ।
প্রাথমিক বিশ্লেষণে তুলনা করা হয় ইউনিয়ন (ক্লাস্টার) স্তরে কন্ট্রোল এলাকার সাপেক্ষে অংশগ্রহণমূলক দলের এলাকা এবং কণ্ঠস্বরের মাধ্যমে বার্তা (ভয়েস এস এম এস) প্রেরিত এলাকার।

ফলাফল
অনির্দিষ্টভাবে বাছাইকৃত ১৬২৩ (৮২%) জনের তথ্য সংগ্রহ করা হয়েছিল এবং ইন্টারমিডিয়েট হাইপারগ্লাইসিমিয়া দল থেকে ১৮১৭ (৮৭%) জনের তথ্য সংগ্রহ করা হয়েছিল। ২০১৮ সালে মোবাইল ফোনের কণ্ঠস্বর বার্তার (ভয়েস এস এম এস) ফলে ডায়াবেটিস সম্পর্কে যে জ্ঞান বৃদ্ধি পেয়েছিল, ২০২১ সালে সেই বৃদ্ধি পর্যবেক্ষণীয় ছিল না। অংশগ্রহণমূলক শিক্ষা ও কর্মের মাধ্যমে যে এলাকাতে কাজ করা হয়েছিল সেই এলাকাতে কন্ট্রোল এলাকার চেয়ে উল্লেখযোগ্যভাবে জ্ঞান বৃদ্ধি পেয়েছিল কিন্ত ইন্টারমিডিয়েট হাইপারগ্লাইসিমিয়া এবং ডায়াবেটিসের প্রাদুর্ভাবের প্রাথমিক ফলাফলে কোন পার্থক্য পরিলক্ষিত হয় নাই (OR অডস রেসিও (৯৫%সিআই) ১.২৩ (০.৮৯, ১.৭০)) অথবা পাঁচ বছরের সম্মিলিত ডায়াবেটিসের ঘটনার ক্ষেত্রেও কোন পার্থক্য পরিলক্ষিত হয় নাই (১.০৪ (০.৭৮, ১.৪০))। অংশগ্রহণমূলক শিক্ষা ও কর্মের মাধ্যমে যে এলাকাতে কাজ করা হয়েছিল সেই এলাকাতে কন্ট্রোল এলাকার চেয়ে উচ্চ রক্তচাপ (০.৭৩(০.৫৪, ০.৯৭)) ও উচ্চ রক্তচাপ নিয়ন্ত্রণের (২.৭৭ (১.৩৪, ৫.৭৫)) ক্ষেত্রে উন্নতি লক্ষ্য করা গেছে।

ব্যাখ্যা
অংশগ্রহণমূলক দলীয় কার্যক্রম শেষ হওয়ার তিন বছর পর ইন্টারমিডিয়েট হাইপারগ্লাইসিমিয়া এবং ডায়াবেটিসের উপর দলের কার্যক্রমের প্রভাব বহাল ছিল না, কিন্ত রক্তচাপের ক্ষেত্রে উপকার লক্ষ্য করা গেছে।

অর্থায়ন
গ্লোবাল এলায়েন্স ফর ক্রনিক ডিজিজেস (জিএসিডি) ডায়াবেটিস এন্ড স্কেল-আপ প্রগামস এর অধীনে মেডিক্যাল রিসার্চ কাউন্সিল ইউকে: এমআর/এমও০১৬৫০১/১ (ডি-ম্যাজিক ট্রায়াল); এমআর/টি০২৩৫৬২/১ (ডিক্লেয়ার স্টাডি)।
