# Supplementary material for: Intermediate hyperglycaemia, diabetes and blood pressure in rural Bangladesh: five-year post-randomisation follow-up of the DMagic cluster-randomised controlled trial
Source: Lancet Reg Health Southeast Asia. 2022 Dec 10;10:100122. doi: 10.1016/j.lansea.2022.100122 (PMC10015271; doi:10.1016/j.lansea.2022.100122)
Supplement: Supplementary Tables [file mmc2.pdf]

Supplementary Table 1 Diabetes, blood pressure, obesity and CVD risk outcome definitions and pre-specified or exploratory nature

**Outcome**		**Definition**	**Pre-specified/ Exploratory**
Blood glucose	Normoglycaemic	Fasting plasma glucose ≤6.0mmol/l	Pre-specified – Primary outcome
	Intermediate hyperglycaemia	Fasting plasma glucose ≥6.1mmol/l to <7.0mmol/l AND<br>two-hour post ingestion of 75g glucose load plasma glucose <7.8mmol/l<br><br>OR<br><br>Fasting plasma glucose <7.0mmol/l<br>AND<br>two-hour post ingestion of 75g glucose load plasma glucose ≥7.8mmol/l to <11.1mmol/l	
	Diabetes	Fasting plasma glucose ≥7.0 mmol/l<br>OR<br>two-hour post ingestion of 75g glucose load plasma glucose ≥11.1mmol/l<br>OR<br>Prior diagnosis of T2DM by a medical professional	
Blood pressure	Hypertension (%)	systolic blood pressure (SBP) ≥ 140 mmHg or a diastolic blood pressure (DBP) ≥ 90 mmHg or current treatment with antihypertensive medication	Pre-specified – secondary outcomes
	Hypertension control (%)	individuals with a medical diagnosis of hypertension having blood pressure measurements below the hypertensive threshold	
Overweight & obesity	Overweight or obese	BMI≥23	
	Abdominal obesity	waist:hip ratio >0·9 for men and >0·85 for women	
Cardiovascular risk	Isolated systolic hypertension	SBP≥140 mmHg and DBP <90 mmHg	Exploratory
	Isolated diastolic hypertension	SBP<140 mmHg and DBP ≥90 mmHg	
	Mean pulse pressure	mean SBP minus mean DBP	

Supplementary Table 2 2021 frequency, proportions and relative (odds ratio) and absolute (coefficient) effects and 95% confidence interval comparing exploratory outcomes adjusted for (1) the stratified, clustered design, (2) the stratified, clustered design and adjustment for household wealth quintile, and (3) (hypertensive outcomes only) the stratified, clustered design and adjustment for household wealth quintile, age group, sex, diabetic status and BMI.

OUTCOMES				Crude[1]	Adjusted[2]	Adjusted[3]
		mHealth	Control	mHealth vs Control	mHealth vs Control	mHealth vs Control
**Walking**	**Participates in brisk walking (%)**	87 (16.5%)	79 (15.4%)	1.17 (0.59, 2.30); p=0.65	1.17 (0.60, 2.26); p=0.65	
	**Median (IQR) time spent brisk walking per week**	120 (60-180)	180 (120-300)	-0.34 (-0.64, -0.05); p=0.024	-0.32 (-0.63, -0.01); p=0.046	
**Diet**	**Mean 24 Hour Dietary Diversity Score (DDS) (sd)**	6.57 (1.84)	6.61 (1.93)	-0.03 (-0.32, 0.26); p=0.86	-0.01 (-0.30, 0.28); p=0.96	
	**No added sugar to foods in previous 24 hours**	173 (39.1%)	154 (37.6%)	1.06 (0.75, 1.50); p=0.74	1.06 (0.75, 1.49); p=0.73	
	**No added salt to foods**	124 (23.6%)	125 (24.4%)	0.95 (0.72, 1.27); p=0.75	0.97 (0.72, 1.29); p=0.81	
	**Mean (sd) monthly household oil consumption**	4.84 (2.05)	4.74 (1.47)	0.11 (-0.17, 0.38); p=0.46	0.14 (-0.15, 0.42); p=0.35	
**Appraisal of Diabetes Scale (ADS)**	**Mean (SD) ADS score among known diabetics (n=97)**	13.9 (4.06)	13.9 (4.93)	0.82 (-2.05, 3.69); p=0.58	1.38 (-1.60, 4.36); p=0.36	
**Depression & anxiety**	**Median (IQR) PHQ2 score (depression screening)**	1 (0-2)	1 (0-2)	0.01 (-0.09, 0.12); p=0.83	0.01 (-0.10, 0.11); p=0.89	
	**Mean (SD) PHQ9 score (among PHQ2 screen positive, n=142)**	11.1 (4.0)	11.1 (4.6)	0.38 (-1.63, 2.38); p=0.71	0.24 (-1.86, 2.35); p=0.82	
	**Median (IQR) GAD7 score (anxiety)**	4 (1-6)	3 (1-6)	0.05 (-0.11, 0.21); p=0.55	0.05 (-0.11, 0.21); p=0.53	
**Blood pressure measures**	**Isolated systolic hypertension***	49 (9.3%)	51 (10.0%)	0.94 (0.60, 1.49); p=0.79	0.95 (0.60, 1.51); P=0.83	0.86 (0.53, 1.39); p=0.53
	**Isolated diastolic hypertension***	10 (1.9%)	15 (2.9%)	0.63 (0.28, 1.41); p=0.26	0.56 (0.24, 1.30); p=0.18	0.53 (0.23, 1.27); p=0.16
	**Mean pulse pressure (sd)**	47.7 (13.6)	48.6 (13.8)	-0.82 (-2.58, 0.94); p=0.36	-0.67 (-2.45, 1.11); p=0.46	-1.12 (-2.63, 0.39); p=0.15

Supplementary Table 3 Additional blood pressure measures in DMagic endline (2018) survey shows no notable differences between PLA and control.

Blood pressure-related measures			Crude[1]	Adjusted[2]	Adjusted[3]
	**Community PLA**	**Control**	**PLA vs Control**	**PLA vs Control**	**PLA vs Control**
**Isolated systolic hypertension***	504 (13.3%)	469 (12.3%)	1.07 (0.85, 1.35); p=0.55	1.05 (0.83, 1.34); p=0.68	1.04 (0.08, 1.36); p=0.75
**Isolated diastolic hypertension***	70 (1.8%)	68 (1.8%)	1.05 (0.68, 1.63); p=0.81	1.04 (0.66, 1.64); p=0.86	1.04 (0.66, 1.63); p=0.86
**Mean pulse pressure (sd)**	51.5 (14.4)	51.5 (15.2)	0.06 (-1.24, 1.35); p=0.93	-0.04 (-1.35, 1.27); p=0.95	0.03 (-1.23, 1.30); p=0.96

*** Denominator is all non-cases**
